# The use of outcome and process indicators to incentivize integrated care for frail older people: a case study of primary care services in Sweden

**DOI:** 10.5334/ijic.1680

**Published:** 2014-12-15

**Authors:** Anders Anell, Anna H. Glenngård

**Affiliations:** Lund University School of Economics and Management, Lund, Sweden; Lund University School of Economics and Management, Lund, Sweden

**Keywords:** integrated care, primary care, frail elderly, incentives, outcome indicators, process indicators

## Abstract

**Background:**

A number of reforms have been implemented in Swedish health care to support integrated care for frail older people and to reduce utilization of hospital care by this group. Outcomes and process indicators have been used in pay-for-performance (P4P) schemes by both national and local governments to support developments.

**Objective:**

To analyse limitations in the use of outcome and process indicators to incentivize integrated care for elderly patients with significant health care needs in the context of primary care.

**Method:**

Data were collected from the Region Skåne county council. Eight primary care providers and associated community services were compared in a ranking exercise based on information from interviews and registered data. Registered data from 150 primary care providers were analysed in regression models.

**Results and conclusion:**

Both the ranking exercise and regression models revealed important problems related to risk-adjustment, attribution, randomness and measurement fixation when using indicators in P4P schemes and for external accountability purposes. Instead of using indicators in incentive schemes targeting individual providers, indicators may be used for diagnostic purposes and to support development of new knowledge, targeting local systems that move beyond organizational boundaries.

## Introduction

Improved integrated care for frail older people with multiple chronic conditions is a central challenge facing health systems globally [1, 2]. An important objective is to maintain or improve health status among the elderly, thereby reducing avoidable acute care episodes and hospitalization. A strong and coordinating role for primary care including continuity in contacts with patients is believed to be necessary to reach the objectives [3, 4]. Preventive measures based on early symptoms and considering individual patients overall situation can then be initiated. Primary care providers need to integrate their services and share information with other providers. Collaboration and coordination with community services and hospitals enable improved case-finding and case-management.

In a recent literature review, 35 publications were identified that studied the association between elderly patients with multiple chronic conditions and health care expenditures [5]. Several studies found that average health care expenditures increased significantly for every additional disease. A study using data from Medicare in 1999 [6] found that the probability for admittance to in-patient care for ambulatory care sensitive conditions was 99 times higher for elderly patients with four or more chronic conditions, compared to elderly patients without a chronic condition. Per capita Medicare expenditure for patients with four or more chronic conditions was 66 times higher than for elderly people without a chronic condition.

According to previous research, a substantial proportion of hospitalization among elderly patients is a result of suboptimal outpatient care and community services and can be avoided [7–10]. Fragmented services to elderly patients with multiple chronic conditions and poor access to primary care physicians are important explanations behind an excessive use of hospital care [8, 9]. When health care providers work in silos without complete information about the health status and overall situation of the elderly patient, or the interventions prescribed by other health care providers, the risk of suboptimal care and avoidable acute visits and hospitalization related to for example polypharmacy and medication errors increases [10, 11]. Acute visits to hospitals and hospitalization are in itself risk-factors for frail older people [12–14]. Hospitalization may create irreversible functional decline and complications unrelated to the reason for admission. The additional medications prescribed by a specialist following acute visits may increase the risk of drug-related problems [11]. The need for integrated care for frail elderly has become more pronounced as rates of institutionalization have dropped across OECD countries to the favour of home care [7]. A main challenge is then to coordinate health care workers regardless of organizational belongings [15]. A stronger evidence base has also been developed recently regarding interventions which may reduce unnecessary use of acute hospital beds [10].

Given the contemporary focus on governance using performance indicators [16], one option to promote integrated care would be to reward primary care providers based on outcome indicators reflecting utilization of hospital care by frail elderly. A soft form of accountability is transparent comparison across providers and to rely on professional reputation mechanisms for quality improvements. Alternatively, outcome or process indicators may be linked to economic incentives such as pay-for-performance (P4P) schemes [17].

Performance indicators based on outcomes have several benefits in terms of holding providers accountable for quality improvements beyond compliance to evidence-based processes [18, 19]. Major concerns are the need for risk-adjustment, attribution problems and the risk of type I (false positive) and II (false negative) errors when rewarding practices due to randomness [20–22]. These three concerns are of particular relevance for primary care providers and integrated care for the frail elderly. Depending on geographic location of primary care providers, the socio-economic profile of registered individuals will vary. Low socio-economic status in turn is associated with increased bed use [10]. Differences in utilization of hospital care across elderly patients may also be attributed to the quality of community staff providing home care, rather than efforts by primary care. Finally, many individual primary care providers register small sample sizes of frail older people, making assessment of performance uncertain due to randomness.

In summary, variation in utilization of hospital care among frail elderly patients may indicate that quality of primary care, such as continuity towards patients and collaboration and coordination with community services and hospitals, varies. However, variation may also reflect statistical uncertainty and/or non-controllable factors from the perspective of primary care, such as the socio-economic status of patients or the efficiency and quality of care provided by community services and/or hospitals. Ideally, providers should only be held accountable for factors that they are able to control. Thus, any use of outcome indicators to hold primary care providers accountable should consider the need for risk-adjustment, attribution problems and randomness.

From an accountability perspective, it may be preferable to use process indicators to incentivize providers. Examples of a process indicator of potential relevance for frail elderly patients are establishment of individualized care plans, with participation from GPs, home care nurses and patients. Accountability towards process indicators has problems of its own, however. The proven association between process indicators and outcomes may be weak or only indirect [10]. Providers may engage in manipulation and “box-ticking” behaviour and reach defined targets without concern for individual patient needs and end objectives [19, 23, 24]. Providers may also already fulfil evidence-based processes, with only a minority of practices lagging behind [18]. Given such conditions, process indicators may have a greater potential when defining a minimum standard for accreditation of providers, rather than supporting continuing improvements.

In Swedish health care, a number of reforms at both national and regional levels have been implemented to improve health care services provided to frail elderly with significant health care needs. Similar to other countries, focus has been attended at the possibility to reduce expensive use of hospital care through preventive measures, improved community services and individualized care plans. To incentivize improvements among the 21 county councils and 290 municipalities responsible for care to the elderly, both outcomes and process indicators have been used within a national P4P scheme. Whereas county councils are responsible for delivering primary and hospital care to its populations, the municipalities are responsible for home care and institutionalized care to elderly. Both outcome and process indicators have also been used by individual county councils in P4P schemes focusing primary care providers.

There is limited evidence to support or not to support a use of P4P incentives to improve the quality of primary care [25, 26]. This calls for a careful design of incentive schemes including choice of valid and reliable indicators [25, 27]. In this article, limitations in the use of outcome and process indicators to incentives-integrated care for frail older people are studied in the context of Swedish primary care. The article reports findings from a mixed method case study in Region Skåne, Sweden. In particular, we analysed the validity of process indicators and to what extent variation in utilization of hospital care (i.e. outcome indicators) for elderly with significant needs could be attributed to factors that primary care providers are able to control. Finally, we discuss policy implications and possible future developments in the use of outcome and process indicators to improve quality and coordination of care for frail older people.

## Materials and Method

Empirical primary data were collected in two steps. First, a qualitative approach was used in a ranking exercise that tried to assess the quality and coordination of care for a sample of eight primary care providers based on 10 process indicators. Second, data from all 150 primary care units in Region Skåne were analysed in regression models. It should be noted that neither the ranking exercise nor the regression analysis was performed with the intention of supporting the evidence base related to process indicators or utilization of hospital care by frail elderly. The main purpose was instead to analyse limitations when using process and outcome indicators to incentivize primary care providers to improve services to frail older people. Secondary data in the form of articles in the Swedish Medical Journal (Läkartidningen) and government reports were used to support discussion of results.

### The ranking exercise

**Figure 1.**
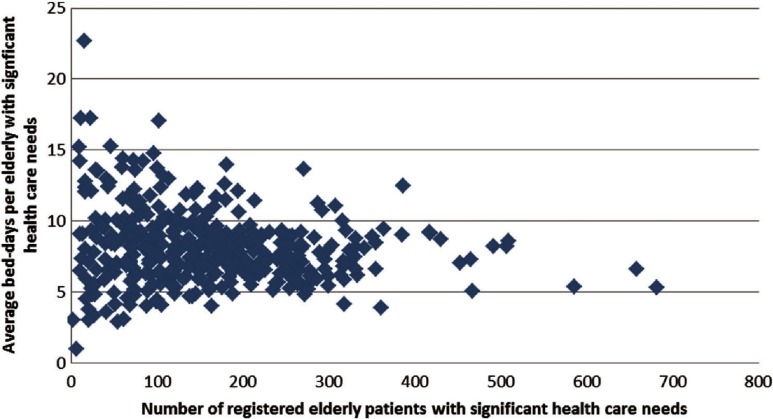
Average number of bed-days for elderly with significant health care needs registered at different primary care practices in Region Skåne, years 2009–2011.

The sample of eight providers in the qualitative approach was selected based on several criteria. First of all, we included providers who reported either high or low utilization of hospital care, measured by average number of bed-days for elderly patients with significant health care needs. Elderly patients with significant health care needs were defined as all patients belonging to the 1.7% of the 1.25 million population in Region Skåne with most significant health care needs measured by adjusted clinical groups, excluding patients <75 years of age. The adjusted clinical group system classifies patients by expected health care utilization in primary care based on diagnosis [28] and is used in Region Skåne as a basis for capitation payment to primary care. Providers who registered less than 150 elderly patients with significant health care needs were excluded from the sample. This exclusion was based on a funnel plot of all practices in Region Skåne years 2009–2011 (see Figure 1) showing that values on both side of the average for the outcome indicator were more spread out for smaller practices. We also selected providers in different geographical districts and in both rural and urban areas. Selected providers are fairly large (between 8000 and 14000 registered individuals) and seven are publicly owned. All primary care providers except one had an agreement with at least two nursing homes to provide medical support to the community care staff. Selection of participating primary care practices was conducted by one of the authors (AA) with support from a medical advisor in Region Skåne [29]. All invited practices accepted to participate in the study.

Information regarding 10 process indicators representing five dimensions of quality and coordination of care was collected using interviews with staff and registered data. Indicators and dimensions had a particular focus on case-finding and case-management and coordination and collaboration between primary care and community services to support integrate services. Interviews with GPs and nurses in managing positions in primary care were matched with interviews with nurses at nearby community services to support triangulation and to gain additional information from a community perspective. Respondents were asked to provide examples where possible. In total, 24 semi-structured interviews were conducted by one of the authors (AHG) without prior knowledge about the level of utilization of hospital care among the eight providers. This principle of “blinded” interviews was performed to prevent bias in the collection and comparison on qualitative data. All interviews were audio taped enabling review by the co-author.

Data from interviews were complemented with registered data used within a P4P scheme in primary care regarding continuity of care, number of medicines reviews to elderly 75+ and number of individualized care plans for elderly in home care. Based on the performance of these and other indicators, primary care providers in Region Skåne were able to generate up to 3% additional payments in 2012.

Based on the collected data, differences between the eight practices were analysed in a ranking exercise. For each indicator, providers were assessed from one to three, where a higher value indicated a favourable position compared to other providers in the sample. The final assessment for each practice was communicated to interviewed GPs and nurses, who had the opportunity to comment and add or clarify information. Overall, the final assessment was accepted.

Ranking of providers was compared with data from two outcome indicators reflecting utilization of hospital care: average number of bed-days for patients 75+ with significant needs, and number of visits to hospital emergency units without hospitalization for patients 75+. These outcome indicators were used in Region Skåne for comparison of different primary care providers. One of them (number of visits) was used within the P4P scheme.

### Regression models

A quantitative approach was used to analyse the association between indicators of hospital utilization (outcome indicators) and a number of factors among all 150 primary care providers in Region Skåne reflecting both process indicators and non-controllable variables from the perspective of primary care providers. Registered data from year 2012 were used in step-wise regression modelling. The same outcome indicators as in the ranking exercise were used as dependent variables:

- average number of bed-days for patients 75+ with significant health care needs;
- number of visits to hospital emergency units without hospitalization per 1000 patients 75+.

Average, rather than median, in-patient days were used as most patients had not been admitted to hospital in-patient care at all. As the average is more sensitive to outliers, patients with 100 or more bed-days during the year were omitted from the analysis. Data related to avoidable hospitalization based on relevant diagnoses for this selected group of frail elderly patients were not available.

Independent variables were chosen from registered data reflecting the same process indicators that were used in the qualitative study. An additional variable reflecting proportion of direct admissions to hospital wards (i.e. side-stepping the emergency intake at hospital) was also included. Most practices used this option to a limited degree and it was not relevant when comparing differences between the eight selected practices in the ranking exercise. Independent process variables were combined with other independent (control) variables reflecting location, ownership, health care needs and socio-economic status of registered individuals across providers. In total, eight independent variables were included in the analysis:

- continuity (proportion of patients meeting the same GP during three consecutive visits);
- proportion of direct admissions from primary care or nursing homes to hospital wards (by-passing the hospital emergency units);
- number of medicines reviews in collaboration with pharmacist and community staff (in relation to number of elderly patients with significant health care needs);
- number of individualized care plans regarding care and social services established in collaboration between primary care and community services (in relation to number of elderly patients with significant health care needs);
- location of provider (five districts with different community and hospital services);
- socio-economic status of registered individuals (measured through care need-index, CNI for each provider in May 2012)
- health care needs for registered individuals (measured through adjusted clinical group in May 2012);
- ownership of providers (public or private).

As the analysis was carried out using aggregate data for each provider, the regression model did not include individual data on for example sex and age of patients. Two included variables (number of medicine reviews and individualized plans) were adjusted with respect to number of elderly patients with significant health care needs, which are more relevant for our purposes. The analysis did not include capacity in terms of available beds at the local hospitals or the quality of discharges. Although relevant variables, such information was not available as the responsibilities of local hospitals in terms of acute services and number of specialties vary and as they collaborate by transfer of patients in case beds are not available. However, location of providers in terms of five geographical districts was included to capture possible differences related to hospital care. The simplified regression analysis in this and other respects was deemed satisfactory in view of our main purpose, to analyse limitations when using indicators to incentivize primary care providers to improve integrated care.

The regression analysis was carried out in SPSS (method = enter). The final models were selected based on the value of explained variance of the dependent variable (adjusted *R*^2^), controlled for multicollinearity between independent variables (tolerance values <0.25 and VIF > 4 were not accepted). Independent variables were assessed based on significance level *p* < 0.05.

## Results

### Results from the ranking exercise

Each primary practice provider was ranked according to the sum of the assessment for each indicator, assuming all dimensions to be equally important (see Table 1).

Case-finding was assessed with respect to structure for ensuring that patients with significant health care needs made regular visits to primary care. Two types of criteria were used by the eight providers. The first was based on primary or community care staff's assessment; individuals with limited ability to seek care themselves were scheduled for regular visits. The second was based on age and/or diagnosis; patients above a certain age and/or having a certain diagnosis were scheduled for regular visits. When assessing and grading this dimension and indicator, the relative comprehensiveness of these two criteria across the eight providers was considered.

Continuity towards patients was assessed with respect to patients having one assigned GP and registered data about proportion of patients meeting the same GP during three consecutive visits to primary care. Accessibility for patients was assessed with respect to opening and phone hours, accessibility for acute visits at the primary care practice and accessibility for scheduled and acute visits in regular housing of elderly patients or at nursing homes. Collaboration between primary care and community services was assessed based on accessibility and continuity of staff in community services, personal knowledge about staff within primary care and community services, formal structures for collaboration including regular meetings and available alternatives for community staff to contact GPs.

Coordination of care was assessed based on the number of individual care plans and medicines reviews according to registered data, combined with self-rated level of coordination. As can be noted from Table 1, the self-rated assessment of coordination differed between primary care and community services staff. In all cases except one, interviewed staff from community services rated the level of coordination as lower compared to the level rated by interviewed staff from primary care. The lack of coordination with community staff related to individual needs of patients is also reflected by a low number of individualized care plans among the eight providers. Several GPs and nurses in primary care referred to a lack of time to engage in such activities. Moreover, interviewed staff from community services explained that in cases where individualized care plans have been developed such processes are often initiated by community staff. The level of efforts put into such processes by primary care staff may also vary. Medicines reviews were found to be more common. Several GPs and nurses within primary care pointed out that issues related to medication were believed to constitute the single most important risk factor for elderly with significant health care needs. This view was confirmed by community staff.

As illustrated in Table 1, most providers performed rather well with respect to case-finding, continuity, accessibility and overall collaboration between primary and community care. Few providers, however, performed well with respect to coordination of care based on individual health care needs of elderly patients.

In Table 2, the summarized assessment is compared with data regarding utilization of hospital care and socio-economic status among registered individuals for each primary care practice. There was no pattern of association between rankings and average number of bed-days. The three providers that were ranked highest also performed best with respect to number of visits. These three providers, however, also had a low level of socio-economic deprivation among registered individuals.

### Results from the regression analysis

To further analyse to what extent outcome indictors are influenced by process indicators or non-controllable factors from the perspective of primary care providers, step-wise regression modelling using available data from all 150 primary care providers in Region Skåne was performed. In Table 3, results are presented from two final regression models with number of visits at emergency units without admission and average number of bed-days as dependent variables. In the final model presented with visits to emergency units as the dependent variable, two explanatory variables are included: location (a lower level of visits in geographical district “Mid” and “North East”) and socio-economic status among registered individuals (a higher level if socio-economic deprivation). In the final model with average number of bed-days as the dependent variable, socio-economic status among registered individuals (a higher level if socio-economic deprivation) and proportion of direct admissions to hospital wards (a lower level of bed use with increased proportion) are included and significant. Other process indicators and control variables were not significant in any of the two models and excluded in the final models presented.

## Discussion

The ranking exercise and regression analysis in this article are simplistic but nevertheless informative and adequate given the main purpose of the study. Previously reported limitations when using outcome and process indicators to incentivize providers are well illustrated and highly relevant when supporting integrated care to frail older people.

Our regression models, using available data from all 150 primary care providers, indicated a significant correlation between utilization of hospital care and non-controllable factors from the perspective of primary care providers, such as location of provider and socio-economic status of registered individuals. Model fit for especially average number of bed-days as the dependent variable was poor, i.e. most of the variation could not be explained by the variables included in the model. A possible confounding factor is that providers differ in the intensity of diagnostic labelling, and thereby in the inclusion of elderly with significant health care needs, although there was no indication of systematic differences in this respect in our data. In practice, utilization of hospital care by frail elderly patients is likely to depend on a number of factors not accounted for in our models. These include the case-mix of patients, number of GPs and other primary care staff, capacity of community services and hospitals, overall efficiency and routines of facilities including discharge planning and more detailed information about continuity, collaboration, coordination and information sharing specifically oriented towards elderly with significant health care needs. Even if all these variables had been included, randomness would still be a significant problem, as suggested by our funnel plot of variation in utilization depending on number of frail elderly patients registered at different providers. The point made here is that outcome indicators related to utilization of hospital care by frail elderly are not reliable to use in incentive schemes addressing individual primary care providers, due to problems of risk-adjustment, attribution and randomness.

Differences depending on socio-economic deprivation may be explained by both demand and supply factors. Patients with poor socio-economic status have been found to utilize more bed-days on average [10]. Working conditions within primary care and community services directed to elderly patients may also be less favourable in poorer socio-economic areas [30]. If such indicators are still used in for example a P4P scheme, rewards will to a significant degree reflect the location of units and the socio-economic status of registered patients. Furthermore, rewards will be random as a higher proportion of both high and low outcomes can be expected for providers who register a smaller number of frail older people. Such random patterns can be expected for all outcome indicators with a normal distribution.

With more aggregated data, such as the P4P scheme in place at the national level to county councils in Sweden, outcome indicators may be more reasonable and relevant as problems related to risk-adjustment, attribution and randomness are reduced. Nevertheless, a recent evaluation of the national P4P scheme recommended the government to exclude outcome indicators reflecting hospital utilization even at this level [31]. In spite of several local government efforts, existing knowledge about how to influence hospital utilization by frail elderly was assessed as limited. An alternative approach, suggested by the Swedish National Board of Health and Welfare, would be to focus on avoidable hospitalization in specific diagnoses of particular relevance for elderly patients, e.g. heart failure, pyelitis, chronic obstructive pulmonary disease, angina and diabetes [32]. Such a use of outcome indicators has also been recommended more generally, to reduce the ambiguity and difficulty of interpreting outcomes for heterogeneous patient groups [33]. Outcome indicators focusing on patient groups with more homogeneous problems may also more easily be combined with specific interventions that address known problems. New schemes have recently been developed for patients with heart failure in Region Skåne, with rewards to providers based on reduction in hospital care combined with an intervention that include closer monitoring of the weight of elderly patients in primary care.

With more specific patient groups, however, problems due to small number of patients and randomness will be even more present. Such developments will consequently not result in more reliable indicators when used to incentivize individual primary care providers. The only way to reduce randomness is to increase the volume of data. One possible solution is to evaluate primary care practices based on rolling data from several years. If so, assessment will have to consider the possibility that outcomes is explained by previous routines. Another solution is to include data from several primary care providers, but then the possibility to hold individual providers to account is lost.

The ranking exercise involving eight primary care providers illustrates several problems described previously in the literature concerning the validity, reliability and feasibility of process indicators. The use of process indicators to incentivize collaboration and coordination, instrumental when developing integrated care, seems particularly troublesome. Our interviews indicated that primary care and community services had consistently different opinions regarding the degree of coordination. Community staff reported a lower degree of coordination based on individual patient needs than primary care staff did. The risk of “box-ticking” behaviour and measure fixation seems particularly relevant since the quality of processes, not only their number, is of great importance.

One example is the use of “number of individualized care plans” to reward primary care providers. As staff from community services explained, such activities are often initiated by themselves and the interest from primary care is weak. The level of effort by primary care staff once individual plans are being developed varies. The direct benefits of individual patient plans are also to be found in hospital care and community services, rather than in primary care. In practice, primary care providers may reach the defined targets without a committed concern for the end objectives. The number of plans is not necessarily a valid or reliable measure of the quality and coordination of care. Medicines reviews were found to be more common, accepted and initiated by primary care. Drug-related problems were identified as an important risk factor for elderly patients by GPs. As primary care providers have a financial responsibility for prescribed drugs, medicines reviews are also associated with economic incentives and direct benefits in terms of reduced costs for prescription drugs, beside the extra payment for each review.

An alternative approach, rather than rewarding the volume of medicines reviews and individual patient care plans, would be to address the end objectives more specifically. Instead of paying for medicines reviews, primary care providers may be rewarded if certain drug-combinations and polypharmacy can be avoided. To accomplish such ends, a medicines review will be required, but it is the end objective rather than the process that is rewarded. For individual patient care plans, the end objective is that GPs, community staff and patients with relatives should know in advance what to do when different symptoms occur. A complementary way of supporting such plans is to measure if such knowledge exists using surveys. Surveys indeed provide more subjective data on the level of coordination and integrated care. It may nevertheless be a more valid measure than if only the volume of activities is accounted for without concern for quality.

Based on findings in this article, one should not be too optimistic about the use of indicators reflecting utilization of hospital care or the number of coordination activities in incentive schemes to support integrated care for frail older people at the provider level. Problems related to risk-adjustment, attribution, randomness, measure fixation and box-ticking behaviour without concern for end objectives are too significant. The evidence based on interventions such as outpatient medicines reviews are also still weak, although several studies have shown an association between polypharmacy and increased utilization of hospital care by older people [10]. Given these problems, alternative strategies for at least the immediate future need to be considered.

Instead of using indicators in incentive schemes and for accountability purposes, an alternative is to apply a diagnostic purpose and to use indicators to support development of knowledge and a stronger evidence base [34]. The main focus in comparisons across providers would then be to identify positive and negative outliers and collect additional data that might explain these and develop new knowledge in targeted studies if needed. For example in one of our regression models, a higher proportion of direct admissions were found to be associated with a lower number of bed-days. This finding is interesting, but further studies are needed to specifically address this co-variation and how it can be explained. In principle, direct admission may cause a lower level of confusion as elderly can by-pass emergency units, thereby reducing length of stay. The possibility to by-pass emergency units at hospitals may also indicate a closer collaboration between primary, community and hospital care. Such collaboration may result in a greater confidence among not least community staff that hospital services can be used if needed, with the end result being a lower use of hospital care. Without additional data and studies, it will not be possible to explain the relationship and to develop effective interventions that can be used by other providers.

Using outcome and process indicators for diagnostic purposes and to develop a stronger evidence base is likely to have a significant and important effect on how the use of indicators is perceived by providers. The use of register data and in particular utilization of hospital care by elderly in P4P schemes have been criticized by Swedish GPs [35, 36].

The experienced reader may rightly conclude that indicators have always been used to support continuous improvement and a scientific interest in effective interventions. The use of indicators in incentive schemes and for accountability purposes is a rather new phenomenon. Our suggestion to use indicators for diagnostic purposes can from this perspective be described as a case of “back to the future”. The use of indicators is however most effective when integrated into a system of continuous monitoring and improvements [37]. In this respect there is also a need for improvements. To support integrated care for frail older people, the system and indicators need to be defined beyond organizational boundaries, rather than focusing on activities performed by individual providers. Evidence also suggest that audit and feedback are most effective if provided regularly in both verbal and written form, if the person responsible is a trusted supervisor or colleague, and if linked to an action plan [38].

Previous studies indicate that factors such as organizational culture and quality of leadership are important for any progress towards improved collaboration between health professions [39]. Accountability towards leadership and governance that supports such an end is consequently important. Incentives to improve integrated care at the provider level may have limited effects without a strong administrative support that enables information sharing and focus on cost-effectiveness rather than separate provider budgets. Such a support can only be developed at a higher administrative level. The national P4P scheme seems to have contributed to an increased interest in integrated care among local governments in the form of county councils and municipalities [31]. The challenge to develop integrated care beyond organizational boundaries at the provider level remains.

## Figures and Tables

**Table 1.**
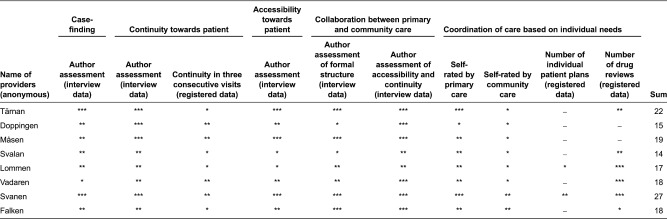
Assessment of quality of care including aspects of coordination and collaboration between primary and community care

**Table 2.**
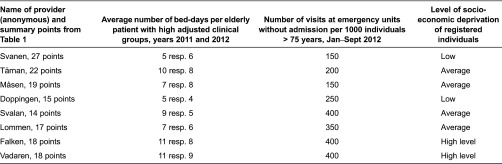
Comparison between ranking exercise, hospital utilization indicators and status of registered individuals

**Table 3.**
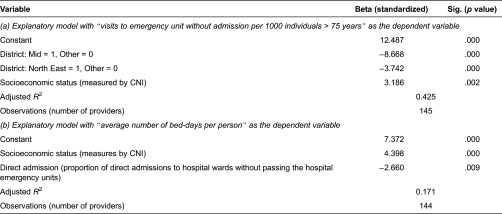
Results from regression models

## References

[1] Singer SJ, Burgers J, Friedberg M, Rosenthal MB, Leape L, Schneider E (2011). Defining and measuring integrated patient care: promoting the nest frontier in health care delivery. Medical Care Research and Review.

[2] Schoen C, Osborn R, Squires D, Doty M, Pierson R, Applebaum S (2011). New 2011 Survey of patients with complex care needs in 11 countries finds that care is often poorly coordinated. Health Affairs.

[3] Saltman RB, Rico A, Boerma W (2006). Primary care in the driver′s seat? Organizational reform in European primary care.

[4] WHO (2008). The World Health Report 2008: Primary health care: now more than ever.

[5] Lehnert T, Heider D, Heinrich S, Corrieri S, Luppa M, Riedel-Heller S (2011). Review: health care utilization and costs of elderly persons with multiple chronic conditions. Medical Care Research and Review.

[6] Wolff JL, Starfield B, Anderson G (2002). Prevalence, expenditures, and complications of multiple chronic conditions in the elderly. Archive of Internal Medicine.

[7] Johri M, Beland F, Bergman H (2003). International experiments in integrated care for the elderly: a synthesis of the evidence. International Journal of Geriatric Psychiatry.

[8] Rizza P, Bianco A, Pavia M, Angelillo IF (2007). Preventable hospitalization and access to primary health care in an area of Southern Italy. BMC Health Services Research.

[9] Ouslander JG, Lamb G, Perloe M, Givens JH, Kluge L, Rutland T (2010). Potentially avoidable hospitalization of nursing home residents; frequency, causes, and costs. Journal of the American Geriatrics Society.

[10] Philp I, Mills KA, Thanvi B, Ghosh K, Long JF (2013). Reducing hospital bed use by frail older people: results from a systematic review of the literature. International Journal of Integrated Care.

[11] The Swedish Council on Technology Assessment in Health Care (2009). Äldres läkemedelsanvändning. Hur kan den förbättras?.

[12] Gillick MR, Serrell NA, Gillick LS (1982). Adverse consequences of hospitalisation in the elderly. Social Science and Medicine.

[13] Creditor MC (1993). Hazards of hospitalization of the elderly. Annals of Internal Medicine.

[14] Graf C (2008). Functional decline in hospitalized older patients. American Journal of Nursing.

[15] Petrakou A (2009). Integrated care in the daily work: coordination beyond organizational boundaries. International Journal of Integrated Care.

[16] Klazinga N, Fischer C, Asbroek A (2011). Health services research related to performance indicators and benchmarking in Europe. Journal of Health Services Research & Policy.

[17] Smith PC, Anell A, Busse R, Crivelli L, Healy J, Karin A (2012). Leadership and governance in seven developed health systems. Health Policy.

[18] Bhattacharyya T, Freiberg AA, Metha P, Katz JN, Ferris T (2009). Measuring the report card: The validity of pay-for-performance metrics in orthopedic surgery. Health Affairs.

[19] Ryan AM, Burgess JF, Tompkins CP, Wallack SS (2009). The relationship between Medicare's process of care quality measures and mortality. Inquiry.

[20] Petersen LA, Woodard LD, Urech T, Daw C, Sookanan S (2006). Does pay-for-performance improve the quality of health care?. Annals of Internal Medicine.

[21] Davidson G, Moscovice I, Resmus D (2007). Hospital size, uncertainty, and pay-for-performance. Health Care Financing Review.

[22] Robinson JC, Williams T, Yanagihara D (2009). Measurement of and reward for efficiency in California's pay-for-performance program. Health Affairs.

[23] Maisey S, Steel N, Marsch R, Gilliam S, Fleetcroft R, Howe A (2008). Effects of payment for performance in primary care: qualitative interview study. Journal of Health Services Research & Policy.

[24] Campbell SM, McDonald R, Lester H (2008). The experience of pay-for-performance in English family practice: a qualitative study. Annals of Family Medicine.

[25] Scott A, Sivey P, Ait Ouakrim D, Willenberg L, Naccarella L, Furler J (2011). The effect of financial incentives on the quality of health care provided by primary care physicians. Cochrane Database of Systematic Reviews.

[26] Eijkenaar F, Emmert M, Scheppach M, Schöffski O (2013). Effects of pay for performance in health care: a systematic review of systematic reviews. Health Policy.

[27] Van Herck P, De Smedt D, Annemans L, Remmen R, Rosenthal MB, Sermens W (2010). Systematic review: Effects, design choices, and context of pay-for-performance in health care. BMC Health Services Research.

[28] Starfield B, Weiner J, Mumford L, Steinwachs D (1991). Ambulatory care groups: a categorization of diagnoses for research and management. Health Services Research.

[30] Steen Carlsson K (1999). Equality of access in health care. Lund Economic Studies no. 86. Diss.

[31] Statskontoret (2013). Sammanhållen vård och omsorg om de mest sjuka äldre. Uppföljning av överenskommelsen mellan regeringen och SKL. Delrapport 4.

[32] Socialstyrelsen (2012). Sammanhållen vård och omsorg om de mest sjuka äldre 2012-Bedömning om kommuner och landsting uppnått grundläggande krav och resultat enligt överenskommelsen mellan regeringen och SKL.

[33] Smith P, Mossialos E, Papanicolas I, Leatherman S, Smith P, Mossialos E, Papanicolas I, Leatherman S (2009). Conclusions. Performance measurement for health system improvement. Experiences, challenges and prospects.

[34] Lilford R, Hohammed MA, Spiegelhalter D, Thomson R (2004). Use and misuse of process and outcome data in managing performance of acute medical care: avoiding institutional stigma. The Lancet.

[35] Järhult B, Engström S, Lindström K (2008). Kan kvalitetsregister värdera vårdkvalitet?. Läkartidningen.

[36] Ljung R (2012). Nej till undvikbar slutenvård som prestationsersättning. Läkartidningen.

[37] Braspenning J, Hermens R, Calsbeek H, Grol R, Wensing M, Eccles M, Davis D (2013). Quality and safety of care: the role of indicators. Improving patient care: the implementation of change in health care.

[38] Ivers N, Jamtvedt G, Flottorp S, Young JM, Odgaard-Jensen J, French SD (2012). Audit and feedback: effects on professional practice and healthcare outcomes. Cochrane database of Systematic Reviews.

[39] Deneckere S, Robyns N, Vanhaecht K, Euwema M, Panella M, Lodewijckx C (2011). Indicators for follow-up of multidisciplinary teamwork in care processes: results of an international expert panel. Evaluation & the Health Professions.

